# Correction: Zheng, N.; Wu, D.; Sun, P.; Liu, H.; Luo, B.; and Li, L., Mechanical Properties and Fire Resistance of Magnesium-Cemented Poplar Particleboard. *Materials*, 2019, *12*, 3161

**DOI:** 10.3390/ma13010115

**Published:** 2019-12-26

**Authors:** Nihua Zheng, Danni Wu, Ping Sun, Hongguang Liu, Bin Luo, Li Li

**Affiliations:** College of Material Science and Technology, Beijing Forestry University, Beijing 100083, China; zheng_nihua@163.com (N.Z.); 15393366580@163.com (D.W.); sp1996sghr@163.com (P.S.); luobincl@bjfu.edu.cn (B.L.); bjfulili@126.com (L.L.)

The authors wish to revise the affiliation, due to the errors regarding affiliation in this published paper [1]. The previous affiliation is shown below:

Wooden Material Science and Application, Beijing Forestry University, Beijing 100083, China

The authors would like to apologize for any inconvenience caused to the readers by these changes.
